# Development of the Malocclusion Impact Questionnaire (MIQ) to measure the oral health-related quality of life of young people with malocclusion: part 1 – qualitative inquiry

**DOI:** 10.1080/14653125.2015.1114712

**Published:** 2016-01-08

**Authors:** Neil Patel, Samantha J. Hodges, Melanie Hall, Philip E. Benson, Zoe Marshman, Susan J. Cunningham

**Affiliations:** ^a^ Eastman Dental Hospital, UCLH Foundation Trust, UK; ^b^ Academic Unit of Dental Public Health, School of Clinical Dentistry, University of Sheffield, UK; ^c^ Academic Unit of Oral Health and Development, School of Clinical Dentistry, University of Sheffield, UK; ^d^ Orthodontic Department, University College London Eastman Dental Institute, UK

**Keywords:** Impact, malocclusion, oral health quality of life, orthodontics

## Abstract

**Objectives**: To seek the views of adolescents with malocclusion about how the appearance and arrangement of their teeth affects their everyday life and to incorporate these views into a new Malocclusion Impact Questionnaire (MIQ). **Methods**: Semi-structured interviews were undertaken with a purposive sample of 30 young people (10–16 years) referred for orthodontic treatment to two dental teaching hospitals. The interviews were recorded, transcribed and analysed using framework analysis. Several themes and sub themes were identified and these were used to identify items to include in the new measure. **Results**: Three themes emerged which were: concerns about the appearance of their teeth, effect on social interactions and oral health/function. Participants expressed the view that their teeth did not look normal, causing them embarrassment and a lack of confidence, particularly when they were with their peers or having their photograph taken. Concerns regarding the potential effect of a malocclusion on oral health, in terms of food becoming stuck between crooked teeth, interferences when chewing and increased risk of damaging the teeth were also identified. The themes were used to generate individual items for inclusion in the questionnaire. **Conclusions**: Common themes relating to the impact of malocclusion on the lives of young people were identified and generated items for the new MIQ to measure the oral health-related quality of life of young people with malocclusion. Part 2 outlines the further development and testing of the MIQ.

## Introduction

Changes in the UK National Health Service over the past 20 years have highlighted the importance of delivering high quality care with input from patients. Successive policies have given increasing emphasis on improving quality of care (DoH, 1997, 2000) and evaluating the success of treatments from the patient's perspective with patient-reported outcomes or PROs (DoH, 2008).

PROs are assessed using measures (PROMs) that may be described as ‘ … any report coming directly from patients about how they function or feel in relation to a health condition and its treatment, without interpretation of the patient's responses by a clinician or anyone else’ (Cochrane Patient-Reported Outcomes Methods Group). The Department of Health introduced national data collection using PROMs in 2009 and all patients in the UK undergoing hip or knee replacements, hernia surgery and varicose vein surgery now complete a short questionnaire before and after surgery. PROMs focus on patient-related issues, such as pain, function and mobility, as indicators of successful treatment; factors which are clearly important to the patient's quality of life (QoL) and health-related quality of life (HRQoL). NHS data are collected using both a generic measure of HRQoL, as well as a measure designed to assess the problems that are specific to the patient's condition.

Historically, the outcome of the orthodontic treatment has been determined using normative measures, developed from the clinicians’ perspective and focussing on clinical outcomes, such as Andrew's six keys of occlusion and the Peer Assessment Rating (PAR) (Richmond *et al*., 1992). However, young people and adults usually seek orthodontic treatment for functional and aesthetic worries related to their teeth and how this might affect their interaction with other people and their community. The importance of considering these concerns in patients referred for orthodontic treatment should not be underestimated (de Oliveira *et al*., 2004; O'Brien *et al*., 2007).

The concept of oral health-related quality of life (OHRQoL) attempts to address social and/or emotional concerns, as well as any symptoms or functional problems from the patient's viewpoint. Locker and Allen define OHRQoL as ‘the impact of oral disorders on aspects of everyday life that are important to patients and persons, with those impacts being of sufficient magnitude, whether in terms of severity, frequency or duration, to affect an individual's perception of their life overall’ (Locker *et al*., 2007).

A number of measures have been designed to assess the impact of oral conditions on children (Jokovic *et al*., 2002; Gherunpong *et al*., 2004; Broder *et al*., 2007). Several studies, using these generic measures of OHRQoL, have shown that malocclusion has an effect on the everyday life and activities of young people (Kok *et al*., 2004; O'Brien *et al*., 2006, 2007); however, two systematic reviews suggested that the association was modest (Liu *et al*., 2009; Dimberg *et al*., 2015). The majority of health or OHRQoL measures cannot be readily applied to orthodontic patients, as they focus on pathological conditions, disease, pain and discomfort. It is widely accepted that orthodontics does not fit the conventional ‘health model’, as the majority of treatment is not related to disease and instead aims to correct a malocclusion against a perceived societal norm (O'Brien *et al*., 1998).

Generic measures are useful for comparisons of OHRQoL between different conditions; however, their meaning and significance have been questioned (Locker *et al*., 2007). Marshman *et al*. (2010) carried out a qualitative study involving young people with malocclusion to explore the face and content validity of one generic measure designed to assess OHRQoL in children, the 16-item short form of the Child Perceptions Questionnaire (CPQ_11–14_-ISF16). They found concerns about several aspects of the measure, including the response format, the use of ‘double’ questions and the interpretation of certain words. Some questions were considered by the young people not to be relevant to the impact of malocclusion and several areas of daily life thought to be relevant were not included. The authors concluded that further consideration should be given to the need for a child-centred malocclusion-specific measure of OHRQoL. Condition-specific measures focus on the particular problems relevant to a disease or disorder, making them more sensitive (Bernabe *et al*., 2008), more acceptable to participants and, therefore, higher completion rates are more readily achievable. Their specific nature makes them more likely to respond to change (Robinson *et al*., 2002).

The aim of this study was to develop a measure capable of capturing the actual and perceived issues, problems, limitations, restrictions and adaptation strategies specific to adolescents with malocclusion. The use of a malocclusion-specific measure will allow clinicians and health policymakers to better understand the effects of malocclusion, and its treatment, on young people over time. The measure might be used as an outcome to assess the benefits of treatment and possibly, combined with a normative needs assessment, to determine treatment need. It is hoped that this, in turn, will lead to the provision of an enhanced quality of care.

Guyatt *et al*. (1986) and Juniper *et al*. (1996) describe the stages that should be followed during the development of HRQoL measures. These include:

- Specifying measurement goals: using descriptors appropriate for measuring OHRQoL in adolescents with malocclusion;
- Item generation: populating the measure with suitable items on the basis of qualitative inquiry;
- Questionnaire formatting: including selecting the appropriate response options, wording and language to avoid leading and biased questions;
- Item reduction: reducing items on the basis of their intensity, frequency and importance;
- Cross-sectional testing to determine initial validity, internal consistency/reliability and test–retest repeatability;
- Longitudinal testing to further test validity and responsiveness.

Part 1 of this report describes the first two aspects of the questionnaire development; specifying measurement goals and item generation. Part 2 will describe the further initial questionnaire development of formatting, item reduction and cross-sectional testing.

## Objectives (Part 1)

- To seek the views of adolescents on the aspects of their malocclusion which affect their everyday life;
- To incorporate these views into a new malocclusion-specific questionnaire.

## Participants and methods

Ethics approval was obtained from the proportionate review sub-committees of Newcastle & North Tyneside Research Ethics Committee (REC reference number 11/NE/0281) and North East – Sunderland Research Ethics Committee (REC Reference 11/NE/0359). All participants were treated according to the principles of the Declaration of Helsinki. Participants signed an assent form and their parent/guardian signing a consent form.

Patients were eligible for inclusion if they were between 10 and 16 years of age, had been referred or accepted for orthodontic treatment, but had not previously undergone any orthodontic treatment. Patients requiring complex multidisciplinary treatment were excluded. Potential participants were identified by the clinician treating them in the Orthodontic Departments at the Eastman Dental Hospital, University College London Hospitals Foundation Trust and the Charles Clifford Dental Hospital, Sheffield Teaching Hospitals NHS Foundation Trust. Further details of the study were then provided by the research team.

Purposive sampling was used, whereby participants were selected in order to ensure representation of the key characteristics which may affect the concepts being explored. Four subcategories were chosen to reflect the influence of age, gender, ethnicity and malocclusion type. Interviews were undertaken by two interviewers (NP and MH). One (NP) was an experienced clinician who underwent training in qualitative interviewing and the other (MH) was an experienced qualitative researcher. Prior to carrying out the interviews, topic guides were developed with reference to the existing literature and the purpose of the study. Due to the child-centred nature of the research, the schedule was not implemented as an exhaustive list of all the aspects to be explored and was used in a flexible manner. As the research progressed, the guide was adapted to explore emergent topics. As new topic areas were raised they were added to the topic guide and discussed with subsequent participants.

The London interviews were carried out in a non-clinical environment with a chaperone present and the Sheffield interviews were undertaken at the participant's home. The interviews were audio-recorded and ranged in length between 5 and 40 minutes. Participants were numbered for confidentiality purposes.

The interviewers transcribed the recordings as soon as possible to ensure that data could be analysed concurrently with data collection. No new data emerged after 15 interviews in each location, at which point the interviews ceased.

A framework approach to analysis was adopted (Ritchie *et al*., 2003). This adhered to the following process:

1. Identification of themes: transcripts from interviews were read and notes were made independently by the two interviewers on the general themes relating to: the impact of malocclusion. Recurrent themes were identified and further developed. Upon development, these themes were organised into themes. An initial thematic framework was constructed by the interviewers and discussed with the study team.
2. Labelling the data: sections of transcripts were labelled by the interviewers to indicate which themes data related to. The thematic framework was refined to include themes initially missed.
3. Sorting the data by theme: Data exploring the same theme were compiled.
4. Data synthesis: Thematic charts were created for each of the main themes retaining the context and language used in the data.
5. Descriptive accounts: The nature and content of each theme was described and the themes discussed within the study team before the themes were finalised.

The measure was constructed based on the themes derived from the framework analysis.

Further development of the questionnaire, including questionnaire formatting, item reduction and cross-sectional testing will be described in Part 2 of this report.

## Results

Thirty participants were interviewed (15 at each centre: Eastman Dental Hospital, 8 males, 7 females; Sheffield 5 males, 10 females) with ages ranging from 10 to 16 years.

Three main themes were identified from the interviews and the results are presented below according to these major concerns:

- Appearance of teeth;
- Effect on social interactions;
- Oral health and function.

### Appearance of teeth

Concerns about dental appearance were a consistent impact of malocclusion. These were related to the position of teeth, particularly the upper incisors, including crowding, increased overjet and diastema. Participants spontaneously described how they would like their teeth to look and what they hoped would be achieved during treatment to reduce this impact. The desired outcome of treatment was typically described in relation to what was perceived to be normal and in terms of their smile.

“I have funny teeth. My teeth are too far forward, it looks goofy … .like the cartoon … .I look silly … just two teeth and they stick out.” (Participant 9, male, aged 11 yrs)

“I don't really like my teeth … they are crooked and horrible … ” (Participant 12, female, aged 14 yrs)

“Like straight and not jutting out … .looking nice and normal … ”. (Participant 1, female, aged 13 yrs)

“I've got a gap between my front two teeth and I don't really like that being there. I just want them closer together and sorted out … I just want them to be normal looking.” (Participant 7, female, aged 12 yrs)

“Umm like if your teeth are all straight, clean and white and not crowded or anything, that's a nice smile.” (Participant 13, male, aged 11 yrs)

“It's not much, it's just everyone has normal teeth, and I don't think they look very nice when I smile” (Participant 24, female, aged 12 yrs)

### Effect on social interactions

This related theme was about the negative emotional state the appearance of their teeth caused young people during social interactions. Negative emotions were evoked by comments about or reactions to their teeth from peers:

“Every so often some people call me ‘bunny rabbit’, it's only every so often and it's not that bad.” (Participant 21, female, aged 11 yrs)

MH: “Was it something you wanted?”

M: “Yeah to stop my friends calling me names.”

MH: “That must be hard.”

M: “I just go along with it, they call me ‘Bugs’, I go along with it, but … Sorting my teeth out so people will stop saying stuff to me, that will make me happy. That gets me down because people say something everyday.” (Participant 27, male, aged 14 yrs)

“Some of them (teeth) are missing and some people laugh. I feel down and sad” (Participant 6, male, aged 11 yrs)

as well as members of their own family:

“It's just that my mum and dad say they are not fine … ”. (Patient 2, male, aged 11 yrs)

“My brother cursed me about my teeth … he said I've got messed up teeth because I have to keep going to the dentist”. (Participant 9, male, aged 11 yrs)

Young people typically described the emotions as embarrassment or self-consciousness. They described how they felt other people were judging them based on their dental appearance and that this was upsetting.

“I feel embarrassed because I've got really bad teeth … really crooked” (Participant 12, female, aged 14 yrs)

“It's really embarrassing everyone looking at my teeth.” (Participant 8, female, aged 13 yrs)

“People judge you when they first meet you because it's one of the most stand out features of your face … its important and it's nice to look professional and generally people like it when you have good teeth … ” (Participant 13, male, aged 11 yrs)

Another specific situation, which triggered this emotional response was having photographs taken.

“I would get really embarrassed having a photo taken because people would know I have funny teeth” (Participant 9, male, aged 11 yrs)

“Upset … .I am not happy when people take photos of me … ”. (Participant 15, female, aged 13 yrs).

Participants described concerns regarding the use of photographs on social media.

“If I didn't know about it and they stuck it out to the big wide world [social media] and everyone could see it I wouldn't like it very much … ” (Participant 13, male, aged 11 yrs).

“I am embarrassed … because of my teeth” (regarding friends tagging pictures of the participant on a social network website. (Participant 3, male, aged 16 yrs).

This emotional response resulted in a range of behaviours from avoiding smiling or laughing in public, at school and/or with friends to avoiding group situations altogether. Young people described how they either hid themselves in group photos or altered their smile to hide their teeth.

“I don't really laugh in groups at school” (Participant 9, male, aged 11 yrs)

“I shut my eyes, so I don't see people looking at me” (Participant 8, female, aged 13 yrs) “I'd like to be more confident when I smile … I wouldn't have to hide away because of my teeth. In my school photo I used to hide away and I wouldn't smile properly … ” (Participant 4, female, aged 12 yrs)

“And like I don't like having my photo taken because like my mouth, my teeth come out. So I smile with it closed and that gives me hamster's cheeks … It's just that they stick out and it's quite hard. I put a lot of make up on so you don't stare at my teeth and look at my eyes” (Participant 21, female, aged 11 yrs)

Participants expressed an opinion that improving their dental appearance would make them more confident and allow them to fit in with their peers more.

“I think I would get more friends … maybe people will like me more. My friends have nice teeth … I'm sort of on the outside … and I would like to fit in more” (Participant 8, female, aged 13 yrs)

“I would be more confident to laugh, happier smiling and enjoy everything more [if my teeth were better” (Participant 9, male, aged 11 yrs)

“It might give me more confidence … maybe talking more in class and in the playground with other people … new people” (Participant 7, female, aged 12 yrs)

“ … .spend more time with friends and people at school … at the moment I tend to go off on my own because I don't fit in” (Participant 8, female, aged 13 yrs)

“So, better teeth might make me feel confident … like in a job interview … or like at a gathering … a party … ” (Participant 12, female, aged 14 yrs)

The effect of dental appearance on social interactions was apparently unrelated to age or gender.

### Oral health and function

A third theme that emerged from the data was concerns, particularly from boys, about the impact of their malocclusion on their dental health. Participants described getting food stuck between crowded teeth and specific aspects of their malocclusion, including interferences in the bite.

“When I bite I have to slide. My bite is too strong as well, my teeth might wear away …  …  … .might wear away and I might have short teeth” (Participant 10, male, aged 13 yrs)

“ … I think it is easier for food to get stuck when teeth are all bunched up and crowded … usually you have to floss after or rinse … ” (Participant 13, male, aged 11 yrs)

“ … some food gets stuck and I have to get my toothbrush right in-between and it hurts … .” (Participant 26, male, aged 13 yrs)

Other concerns included problems biting and chewing specific foods, particularly meat.

“If I want to eat chicken and stuff, I can't eat at the front so my mum cuts it up” (Participant 6, male, aged 11 yrs)

“When I am eating meat … my teeth might touch each other and it sort of gets in the way” (Participant 10, male, aged 13 yrs)

“I'd rather have the teeth right … I can talk normally then” (Participant 4, female, aged 12 yrs)

“Because my teeth aren't in the right place, some are pointing up, some are pointing down, they don't crunch properly … I'm getting braces, so I can get my teeth in place when I chomp … Like straight and gum line straightened and teeth are all straight” (Participant 17, male, aged 11 yrs)

The final issue identified was the perception that there might be an increased risk of trauma to teeth because of their malocclusion. Some participants had experienced dental trauma and wanted orthodontic treatment to deal with the resultant effects, others were concerned that their malocclusion meant the risk of trauma was higher and were worried regarding this.

“I know they [the front teeth] are at risk from getting hurt because I play netball and you might get a netball in your face” (Participant 7, female, aged 12 yrs)

“Something bad might happen … .a major chip … .if I fall over I might accidently crack it” (Participant 10, male, aged 13 yrs)

The quotes and themes were used to generate items for the Malocclusion Impact Questionnaire (MIQ), which were broadly divided into three sections:

- How I feel about the way my teeth look;
- How my teeth affect my life;
- The health of my teeth, including eating and knocks/ bangs to my teeth.

## Discussion

Malocclusion impacts on the daily lives of young people through their concerns about their appearance, which leads to negative emotions and affects their social interactions, as well as some functional impacts on eating. Analysis of the data from semi-structured interviews allowed the perspective of young people to be incorporated into the development of a malocclusion-specific OHRQoL questionnaire. This method of developing the MIQ ensured that it is a patient-centred instrument containing items of importance to the daily lives of young people.

The topic guide was used flexibly to guide the interviews and allowed new topics, which were raised by the participants, to be explored fully. All questions were asked in a neutral manner and in a non-leading way to avoid introducing interviewer bias (Liamputtong, 2013). Additionally, all interviews were carried out in a non-clinical area, usually without the presence of parents or guardians. A sample size of 15 participants was sufficient to reach data saturation at each site. This is similar to other qualitative studies with young people about the impact of oral conditions (Marshman *et al*., 2009).

Young people's thoughts and feelings regarding their teeth were predominantly related to their appearance and particular occlusal features, as previously reported by Klages *et al*. (2004) and Johal *et al*. (2007). Common phrases and responses during the interviews related to the position of the participants’ teeth, with words and phrases such as ‘crooked’, ‘messed up’, ‘ugly’ and ‘not in a straight line’ consistently used. For some young people, the appearance of their teeth had a direct impact on their life when in social environments and, in particular, when interacting with peers. The effects of concerns about the appearance of teeth led some young people to alter the way they smiled or laughed or to withdraw from social groups, which resulted in feelings of isolation. Participants drew upon experiences at school, such as having a group photograph taken, as significant events that triggered concern and led to a self-conscious attitude towards their teeth. Similar findings have previously been described in studies of bullying in orthodontic patients (Seehra *et al*., 2011).

Comments about their teeth from peers and family reinforced the individuals own negative perceptions. Participants suggested that their teeth led to them feeling embarrassed in social situations and uncomfortable with regards to smiling and having their pictures taken. Many participants reported that they were ‘unhappy’ and ‘upset’ when friends and/or family uploaded photographs showing their teeth on social networking sites such as Facebook™. They also indicated that their teeth contributed to negative thoughts and feelings and this state of mind played a pivotal role in some participants avoiding group interaction and affected their ability to make friends. Some said that they felt lonely because of their teeth and this led to avoidance of group communication, as discussed in the previous theme. Seehra *et al*. (2011) found that an unaesthetic dental appearance can have a severe impact on a young person's social well-being, with children being mocked and teased due to their malocclusion. The use of a patient-reported outcome measure to assess changes in social well-being before and after treatment would be beneficial to evaluate the effect of orthodontic treatment at addressing this impact.

The effect of a malocclusion on dental health was a third theme identified. Although there is no strong evidence to suggest that orthodontic treatment can reduce the risk of dental caries and periodontal disease, some participants did discuss difficulty cleaning their teeth due to crowding and had sought treatment to improve dental health. One interesting finding was that some children raised the issue of functional problems due their malocclusion, in terms of difficulties biting or chewing certain foods or an increased risk of trauma. There is limited information about this in the literature and worthy of further investigation. It is possible that young people avoid high risk activities because of the perception that teeth are at greater risk of damage due to their malocclusion. This may have a significant impact on the QoL for these individuals, as it may prevent them taking part in the activities they enjoy.

A limitation of this study is that it was undertaken in two hospital-based orthodontic departments in the UK, where orthodontic treatment is provided free for young people under the National Health Service, as long as they fulfil certain occlusal criteria. These criteria are defined in a treatment priority guide, called the Index of Orthodontic Treatment Need. In addition, the young people had all been referred for orthodontic treatment. Young people who have not been referred for an orthodontic opinion, are not able to be referred or are seeking treatment under other health systems might have different motivations to pursue treatment, which have not been explored in this study.

This child-centred approach allowed identification of the main themes from young people to be used as the basis for questionnaire development. Clearly it is important to establish whether these impacts are affected by orthodontic intervention and this was the primary aim of developing this condition-specific PROM. Following further psychometric testing of the questionnaire, its use in larger studies will allow the opinions of large numbers of patients to be obtained and by assessing outcomes of treatment in this way, future service development can be guided.

## Conclusions

- Through qualitative inquiry three themes relating to the impact of malocclusion on the lives of young people have been identified, namely: the concerns of young people about the appearance of their teeth; the effect of malocclusion on their social interactions and; the influence of the malocclusion on their oral health and function;
- These themes have informed the identification of items for the new Malocclusion Impact Questionnaire (MIQ) to measure the OHRQoL of young people with malocclusion;
- Part 2 of this report will outline the further development and testing of the MIQ, including formatting, item reduction and cross-sectional evaluation.

## Disclaimer statements

**Contributors**: Neil Patel was responsible for the study design, ethical approval (London), data collection, analysis and interpretation, as well as the writing of the report. Samantha Hodges was responsible for the study design, ethical approval, data analysis and interpretation and preparation of the report. Melanie Hall was responsible for the study design, data collection, analysis and interpretation, as well as the preparation of the report. Philip Benson was responsible for study design, ethical approval (Sheffield), data analysis and interpretation, as well as the writing of the report. Zoe Marshman was responsible for the study design, data analysis and interpretation, as well as the preparation of the report. Susan Cunningham was responsible for the study design, ethical approval (London), data collection, analysis and interpretation, as well as the writing of the report. All the authors have seen and approved the final report. Susan Cunningham is the guarantor.

**Funding**: Internal funding from the Orthodontic Department, University College London Eastman Dental Institute and Academic Unit of Oral Health and Development, School of Clinical Dentistry, University of Sheffield.

**Conflicts of interest**: None.

**Ethics approval:** The proportionate review sub-committees of Newcastle & North Tyneside Research Ethics Committee (REC reference number 11/NE/0281) and North East – Sunderland Research Ethics Committee (REC Reference 11/NE/0359).
